# Supplementary material for: An Unusual Two-Domain Thyropin from Tick Saliva: NMR Solution Structure and Highly Selective Inhibition of Cysteine Cathepsins Modulated by Glycosaminoglycans
Source: Int J Mol Sci. 2024 Feb 13;25(4):2240. doi: 10.3390/ijms25042240 (PMC10889554; doi:10.3390/ijms25042240)
Supplement: Supplementary file 1 [file ijms-25-02240-s001.zip › ijms-2796355-supplementary.pdf]

Supplementary Materials

An Unusual Two-Domain Thyropin from Tick Saliva: NMR Solution Structure and Highly Selective Inhibition of Cysteine Cathepsins Modulated by Glycosaminoglycans

Zuzana Matoušková, Katarína Orsághová, Pavel Srb, Jana Pytelková, Zdeněk Kukačka, Michal Buša, Ondřej Hajdušek, Radek Šíma, Milan Fábry, Petr Novák, Martin Horn, Petr Kopáček, Michael Mareš

Supplementary Figures

atg	ctg	aag	tca	agt	ata	gta	gtg	ttt	gct	att	tgt	ttc	gtg	gtt	tac	atc	aac	tgc	gtg	60
M	L	K	S	S	I	V	V	F	A	I	C	F	V	V	Y	I	N	C	V	20
cca	act	cga	gtg	gcc	aac	aat	gga	gct	tcc	cga	ccc	ctg	agt	gac	tgc	gag	caa	cgt	aaa	120
P	T	R	V	A	N	N	G	A	S	R	P	L	S	D	C	E	Q	R	K	40
cag	agg	gag	gaa	cgt	aac	acg	ggt	ccg	ctg	gcc	atc	cgc	atc	gag	tgc	aac	ccc	gat	gga	180
Q	R	E	E	R	N	T	G	P	L	A	I	R	I	E	C	N	P	D	G	60
agc	tac	aag	ccg	atg	caa	tgc	ttt	gga	aat	ccc	gat	caa	ccc	cgc	agg	atg	tgt	gcc	tgc	240
S	Y	K	P	M	Q	C	F	G	N	P	D	Q	P	R	R	M	C	A	C	80
tac	gat	caa	gag	tac	gac	cag	atc	aag	gcc	cca	tcg	agg	cag	ctg	aag	tcc	tgc	aag	tgt	300
Y	D	Q	E	Y	D	Q	I	K	A	P	S	R	Q	L	K	S	C	K	C	100
ctg	gca	gag	cat	cac	gag	aag	tca	aag	tcc	aca	cat	agt	caa	gtt	gga	gac	gac	att	ccc	360
L	A	E	H	H	E	K	S	K	S	T	H	S	Q	V	G	D	D	I	P	120
aag	tgt	aat	ctg	acg	agc	gga	tat	tat	gag	caa	atg	cag	tgc	aac	act	cag	cag	cat	tgg	420
K	C	N	L	T	S	G	Y	Y	E	Q	M	Q	C	N	T	Q	Q	H	W	140
tgc	gtg	gat	cca	gaa	agt	gga	acc	gca	ctt	gga	gaa	agg	cgt	tcc	gga	ggc	tgc	acc	gaa	480
C	V	D	P	E	S	G	T	A	L	G	E	R	R	S	G	G	C	T	E	160
gct	gcg	cgc	gac	cac	tgc	taa														501
A	A	R	D	H	C	*														166

Figure S1. Nucleotide and deduced protein sequence of IrThy. This sequence was deposited in GenBank under accession number PP107940.

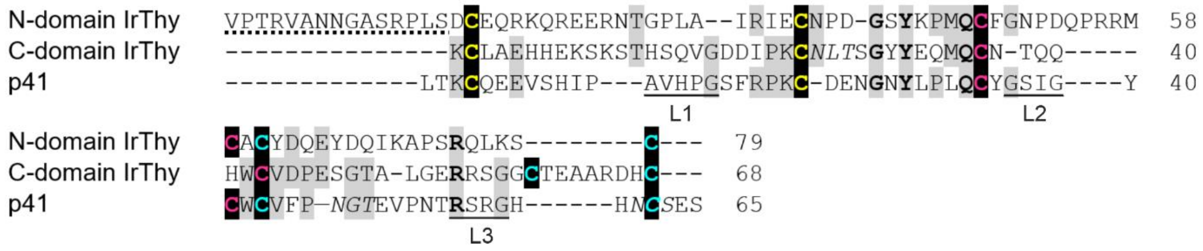

Figure S2. Sequence alignment of the N- and C-domains of IrThy. The human p41 fragment (p41) is included as a prototype member of the thyropin family. The unstructured extension of the N-domain is marked by a dotted line; the N-glycosylation motif is in italics. Cysteine residues forming disulfide bridges are shaded in black; disulfide connectivity is indicated by color coding. Fully conserved residues are in bold and shaded in grey; other identical residues are shaded in grey. The alignment was generated using ClustalQ and edited based on the structural superposition of the IrThy C-domain and p41 (Figure 6B). Residue numbering of IrThy is according to the mature protein. Three regions topped with the binding loops L1–L3 are underlined in the p41 sequence based on the structure of the p41–protease complex (Figure 6D). Sequence comparison shows that the N-domain of IrThy shares the disulfide pattern with p41, while that in the C-domain is modified, and that both IrThy domains differ in two insertions/deletions in the vicinity of the L2 and L3 loops.

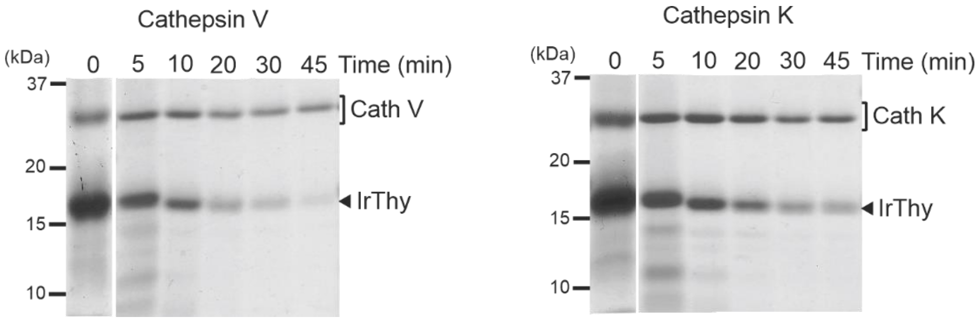

Figure S3. Time course of the proteolytic degradation of IrThy by target cathepsins. IrThy was treated with high concentrations of cathepsins V and K (an enzyme:inhibitor ratio of 1:5, w/w). The reaction mixture was incubated for 45 min at pH 5.5; the aliquots at indicated time points were resolved by SDS–PAGE and visualized by protein staining. The positions of IrThy and cathepsins (Cath V and Cath K) are indicated.

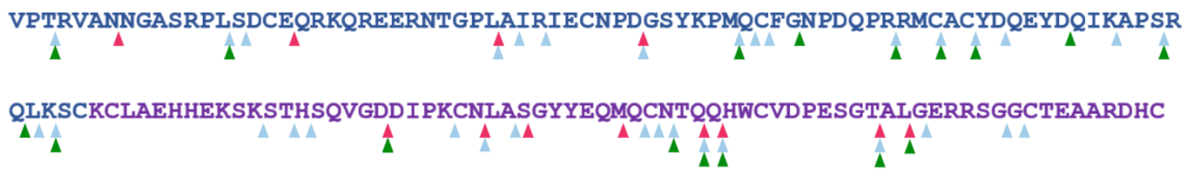

Figure S4. Proteolytic cleavage map of the IrThy molecule. Recombinant IrThy was digested by cathepsin V, K, or L under the same conditions as in Figure 4 at a high enzyme concentration. The fragments were identified by mass spectrometry, and the corresponding cleavage sites are indicated in the IrThy sequence (the N- and C-domains of IrThy are in blue and purple, respectively) as follows: cleavage by cathepsin V (▲), cathepsin K (▲), and cathepsin L (▲). Mass spectrometry analysis of the reaction mixtures after cathepsin digestion was performed as described in the Methods section 4.6. (without additional trypsin digestion).

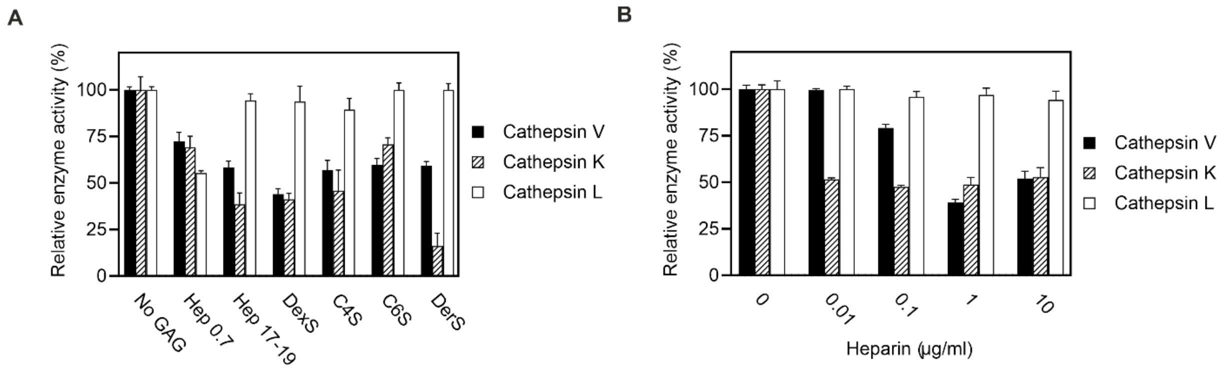

Figure S5. Effect of glycosaminoglycans (GAGs) on human cathepsins V, K, and L (control experiments for Figure 5). The kinetic activity assay with fluorogenic peptide substrates was performed at pH 5.5, and the activity was expressed relative to a control without GAG; means ± SD are given. (A) The following GAGs were used in the assay at 10 µg/mL: heparin (Hep 17-19) and its disaccharide fragment (Hep 0.7), chondroitin-4-sulfate (C4S), chondroitin-6-sulfate (C6S), dermatan sulfate (DerS), and the GAG analog dextran sulfate (DexS). (B) The assay contained various concentrations of heparin (0–10 µg/mL).

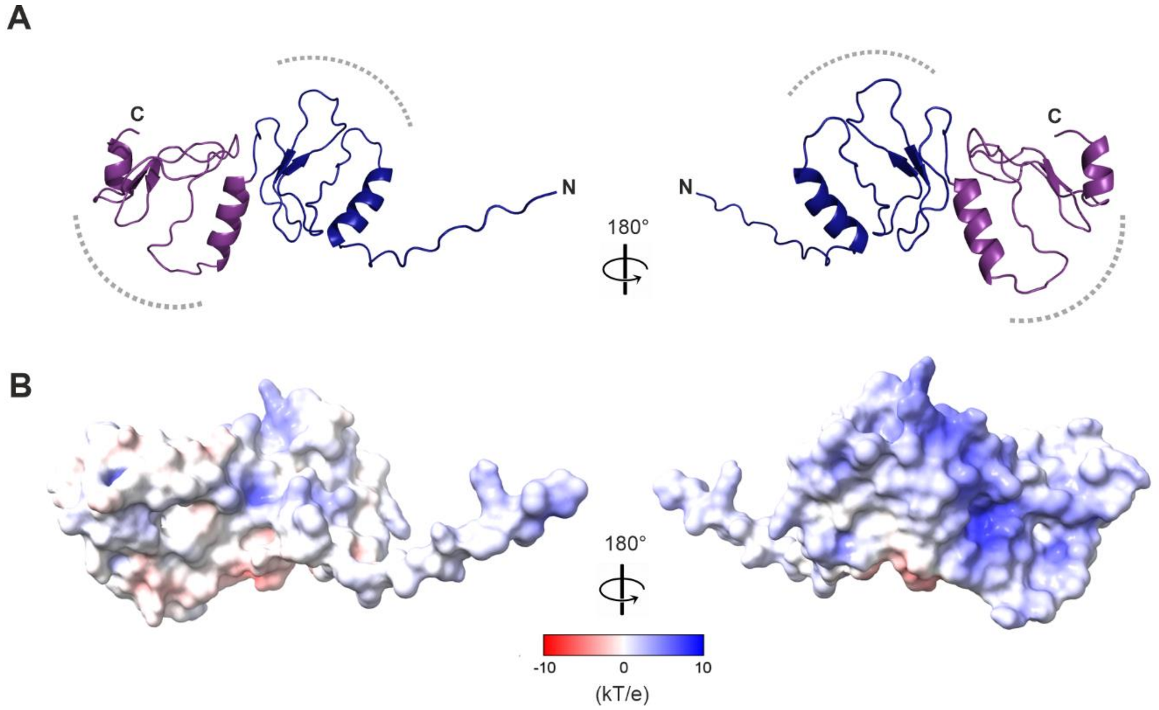

Figure S6. Predicted spatial structure of full-length IrThy. The structure was predicted by AlphaFold2 using the ColabFold web server [1]. (**A**) The model is depicted in a cartoon representation colored by domains: the N-terminal domain is in blue, the C-terminal domain in purple. The N- and C-termini are labeled (N, C); the N-terminus is located on a short unstructured region. On each domain, the dotted line marks three binding loops that form the reactive center involved in the interaction of thyropin inhibitors with cysteine cathepsins [2]. (**B**) Molecular surface of the IrThy model is colored by its electrostatic potential, which is displayed on a scale from −10 kT/e (red) to +10 kT/e (blue). The orientation of IrThy is the same as in (**A**). The electrostatic potential was calculated for pH 5.5 using the APBS-PDB2PQR software suite [3], and the figure was generated using ChimeraX [4].

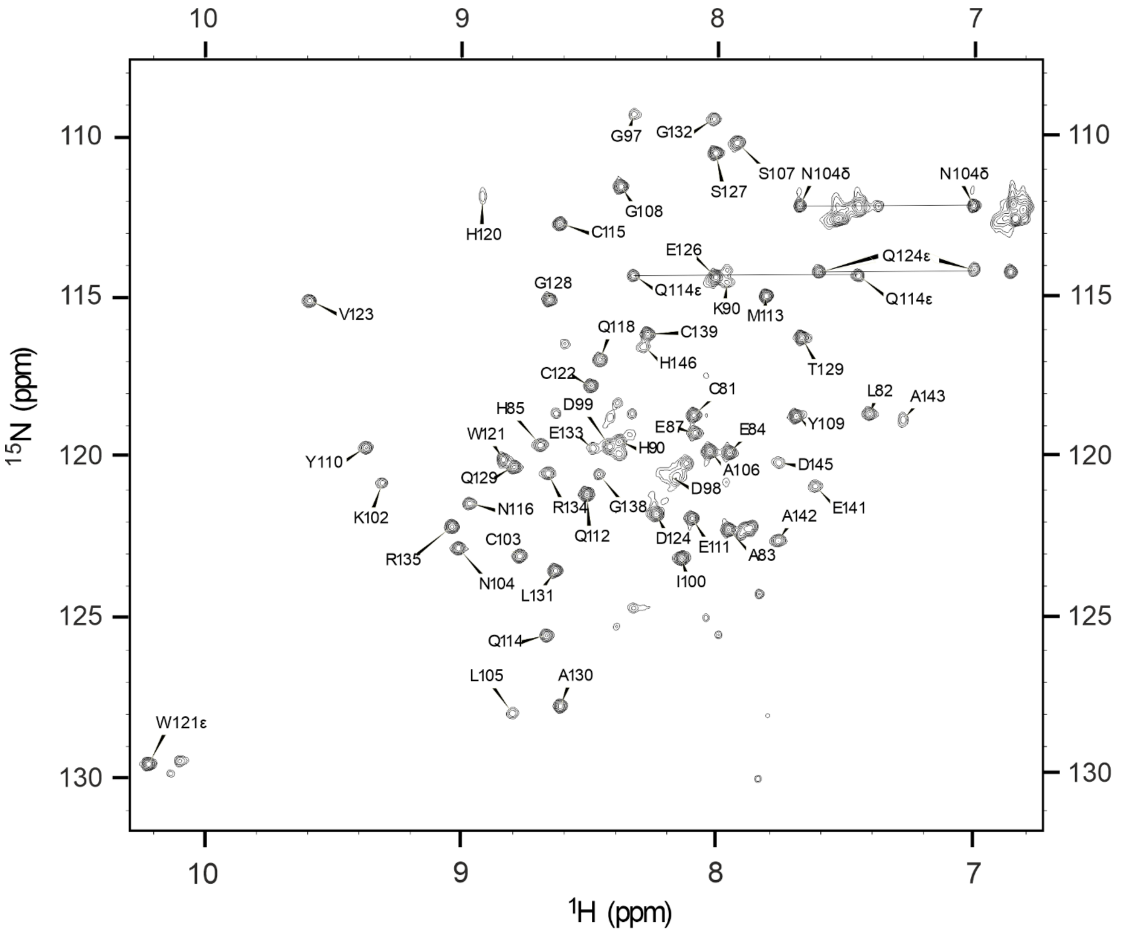

Figure S7. 2D ^{1}H-^{15}N NMR spectrum of IrThy-Cd with the assigned residues indicated. Dotted lines connect signals from the same side-chain amino groups. Residue numbering is according to full-length IrThy.

Supplementary Tables

Table S1. Mass spectrometry identification of native IrThy. Listed are peptides identified in the tryptic digest of proteins from saliva and those extracted from salivary glands of half-fed females of *Ixodes ricinus*. Peptides were analyzed by LC–MS/MS as described in Materials and Methods. Mass deviation (ppm) between experimental and theoretical values and residue numbers of identified peptides are indicated.

Source	Theoretical Mass [M] (Da)	Experimental Mass [M] (Da)	Deviation (ppm)	Peptide Sequence
Salivary gland	2133.901	2133.892	4.22	**MCACYDQEYDQIKAPSR** (58–74)
Salivary gland	2290.004	2290.010	2.62	**RMCACYDQEYDQIKAPSR** (57–74)
Saliva	1722.669	1722.669	0.00	**MCACYDQEYDQIK** (58–70)
Saliva	2133.908	2133.892	7.50	**MCACYDQEYDQIKAPSR** (58–74)
Saliva	1878.772	1878.770	1.07	**RMCACYDQEYDQIK** (57–70)
Saliva	2290.004	2289.993	4.80	**RMCACYDQEYDQIKAPSR** (57–74)

Table S2. Mass spectrometry analysis of disulfide connectivity. Listed are disulfide-bonded peptides identified in proteolytic digests of recombinant IrThy, IrThy-Nd, and IrThy-Cd by LC-MS/MS, as described in a note below the table. Mass deviation (ppm) between experimental and theoretical values, residue numbers of identified peptides and disulfide-bonded cysteine residues are indicated. In the peptide sequences, the disulfide bridges are presented schematically in red. Note that IrThy contains two interdomain disulfide bridges, Cys61-Cys81 and Cys79-Cys103. Residue numbering is according to full-length IrThy.

Experimental Mass [a] [M] (Da)	Theoretical Mass [M] (Da)	Deviation (ppm)	Protein	Connected Peptides	Peptide Sequences	Disulfide Bond
1222.4845	1222.4838	0.57	IrThy–Nd	(16–20)-(35–39)	DCEQR IECNP	C17–C37
2364.8898	2364.8906	0.34	IrThy–Nd	(40–52)-(58–62)-(78–80)	DGSYKPMQCFGNP MCACY SCK	C48–C59; C61–C79
1194.5041	1194.5049	0.67	IrThy–Cd	(81–83)-(103–110)	CLA CNLASGYY	C81–C103
2854.0878	2854.0876	0.07	IrThy–Cd	(111–123)-(136–144)-(145–147)	EQMQCNTQQHWCV SGGCTEAAR DHC	C115–C139; C122–C147
1222.4834	1222.4838	0.33	IrThy	(16–20)-(35–39)	DCEQR IECNP	C17–C37
2333.8845	2333.8848	0.13	IrThy	(40–52)-(58–62)-(81–83)	DGSYKPMQCFGNP MCACY CLA	C48–C59; C61–C81
1224.5027	1224.5035	0.65	IrThy	(78–80)-(103–110)	SCK CNLASGYY	C79–C103
2854.0882	2854.0876	0.14	IrThy	(111–123)-(136–144)-(145–147)	EQMQCNTQQHWCV SGGCTEAAR DHC	C115–C139; C122–C147

[a] Disulfide pairing analysis: The protein sample was digested with Asp-N protease or trypsin (enzyme:protein ratio 1:15, w/w) for 10 h in 50 mM ethylmorpholine pH 8.5, 10% acetonitrile, and 100 μM cysteamine. LC–MS/MS analysis was performed according to a previous protocol [5]. Briefly, the peptide mixture was injected onto a C18 desalting column (Luna Omega Polar C18 5 μm, 0.3 × 30 mm) and then separated on a C18 column (Luna Omega Polar C18, 3 μm, 0.3 × 150 mm) by a linear acetonitrile gradient of 5–35% (v/v) at 50 °C. An Agilent 1290 UPLC system was coupled to the electrospray ionization source of a 15T solariX XR FT-ICR mass spectrometer. The eluted peptides were analyzed in a positive mode with one million transient data points over the 250–2500 *m*/*z* range. The final spectrum was created by averaging the four following spectra with accumulation ions in the collision cell for 0.2 s. The mass spectrometer was operated in data independent mode. Fragmentation data were acquired with 12 eV collision voltage, 0.2 s MS/MS ion accumulation; two scans were accumulated per spectrum. The raw data were processed using Data Analysis 4.4 software. The exported txt files were uploaded into LinX 2.0 [6], and the search was performed with the following setup: protease – AspN or trypsin with two missed cleavages; variable modification – oxidation of methionine, modification of cysteine by cysteamine; disulfide bridge of cysteines; mass error – 1 ppm. All identified peptides with disulfide bridges were checked manually.

Table S3. Mass spectrometry analysis of intermolecular cross-links in the equimolar complex of IrThy with cathepsin V. The chemical cross-linking reaction was performed with disuccinimidyl dibutyric urea (DSBU). Listed are cross-linked peptides identified in the proteolytic digest of the complex by LC–MS/MS, as described in a note below the table. Mass deviation (ppm) between experimental and theoretical values, residue numbers of identified peptides, and cross-linked residues are indicated. In the peptide sequences, the cross-linked residues are in red. The data obtained indicate that the cross-linked residues of IrThy belong to its N-domain. The homology model of the complex of IrThy (bound via its N-domain) with cathepsin V was constructed using an AlphaFold model of IrThy (Figure SX) and the crystal structure of cathepsin V (PDB code: 1FH0); these structures were superimposed on the structure of the complex of the p41 fragment with cathepsin L (PDB code: 1ICF). In the homology model, the identified cross-links are formed between (i) the segments adjacent to the binding loops of IrThy and (ii) the region surrounding the active site of cathepsin V. This result is consistent with the predicted homology model of the IrThy–cathepsin V complex and suggests that IrThy binds preferentially via its N-domain to cathepsin V under equimolar conditions.

Experimental Mass [a] [M] (Da)	Theoretical Mass [M] (Da)	Deviation (ppm)	Connected Peptides	Peptide Sequences	Cross-Linked Residues
5722.7024	5722.700	0.43	CatV (125–159) × IrThy (13–26)	AVATVGPISVAMDAGHSSFQFYKSGIYFEPDCSSK × PLSDCEQRKQREER	S158 × K21
6609.0710	6609.072	0.12	CatV (125–159) × IrThy (57–77)	AVATVGPISVAMDAGHSSFQFYKSGIYFEPDCSSK × RMCACYDQEYDQIKAPSRQLK	K147 × K70
6437.9502	6437.945	0.80	CatV (21–58) × IrThy (58–74)	QCGSSWAFSATGALEGQMFRKTGKLVSLSEQNLVDCSR × MCACYDQEYDQIKAPSR	S24 × K70
7560.5138	7560.512	0.21	CatV (125–159) × IrThy (58–80)	AVATVGPISVAMDAGHSSFQFYKSGIYFEPDCSSK × MCACYDQEYDQIKAPSRQLKSCK	K147 × K77
7720.5163	7720.517	0.13	CatV (148–181) × IrThy (24–56)	SGIYFEPDCSSKNLDHGVLVVGYGFEGANSDNSK × EERNTGPLAIRIECNPDGSYKPMQCFGNPDQPR	K159 × K44

[a] Cross-linking mass spectrometry (XL-MS) method: An equimolar mixture of the IrThy–cathepsin V complex (12 μM, in sodium acetate pH 6.0) was cross-linked using a 100-fold molar excess of DSBU; an active site mutant of cathepsin V was used for this experiment [7]. The cross-linked samples were diluted twice with 100 mM ethylmorpholine pH 8.5 and then reduced and alkylated with 5 mM tris(2-carboxyethyl)phosphine (TCEP) and chloroacetamide (CAA) for 10 min at 70 °C. Subsequently, the samples were digested twice with a combination of trypsin/Lys-C (enzyme:protein ratio 1:20, w/w) for 4 h, followed by the addition of 0.1% TFA. Peptides were analyzed by LC–MS/MS using a Vanquish chromatography system coupled to a timsTOF SCP mass spectrometer equipped with a captive spray source. The peptide mixture was injected onto a C18 trap column (Pepmap Neo C18, 5 μm, 0.3 × 5 mm) and then separated on a C18 column (Pepsep C18, 1.5 μm, 150 × 0.15 mm) by a linear acetonitrile gradient of 5–35% (v/v) at 50 °C. Parameters from the standard proteomics PASEF method were used to set a timsTOF SCP. The target intensity per individual PASEF precursor was set to 20,000 and the intensity threshold was set to 1500. The scan range was set between 0.6 and 1.6 V s/cm^2 with a ramp time of 100 ms. The number of PASEF MS/MS scans was 10. Precursor ions in the *m*/*z* range between 100 and 1,700 with charge states ≥ 2^+ and ≤ 6^+ were selected for fragmentation. The active exclusion was enabled for 24 s. The raw data were processed using Data Analysis 5.2 software and exported to Mascot generic files. The search was performed using Merox 2.0 software [8]with the following setup: protease – trypsin/LysC with three missed cleavages; variable modification – oxidation of methionine, stable modification – carbamylation of cysteine; mass shift for DSBU and reporter ions – 196.085, 85.053 and 111.032; mass error – 10 ppm for precursor and 15 ppm for fragments. All cross-links identified in triplicate were manually checked in the raw spectra.

Table S4. NMR constraints and statistics for the final set of structures of IrThy-Cd.

**Non-Redundant Distance and Angle Constraints**	
Total number of NOE restraints	364
Intraresidue (i = j)	124
Sequential (| i – j | = 1)	108
Medium-range NOEs (1 < | i – j | < 5)	30
Long-range NOEs (| i – j | ≥ 5)	102
Torsion angles	82
Hydrogen bond restraints	32
Total number of restricting restraints	478
Total restricting restraints per restrained residue	8.1
**Residual Constraint Violations**	
Distance violations per structure	
0.1–0.2 Å	0
0.2–0.5 Å	0
> 0.5 Å	0
r.m.s. of distance violation per constraint	0.02 Å
Maximum distance violation	0 Å
Dihedral angle violation per structure	
1–10 °	7.33
> 10 °	0
r.m.s. of dihedral violations per constraint	1.45 °
**Ramachandran Plot Summary**	
Most favoured regions	87%
Additionally allowed regions	13%
Generously allowed regions	0%
Disallowed regions	0%
r.m.s.d. to the mean structure	All / ordered
All backbone atoms	1.4 / 0.9 Å
All heavy atoms	2.0 / 1.3 Å
**PDB Entry**	8R6T
**BMRB Accession Code**	34883

References

1. Jumper, J.; Evans, R.; Pritzel, A.; Green, T.; Figurnov, M.; Ronneberger, O.; Tunyasuvunakool, K.; Bates, R.; Žídek, A.; Potapenko, A.; et al. Highly accurate protein structure prediction with AlphaFold. *Nature* **2021**, *596*, 583-589, doi:10.1038/s41586-021-03819-2.
2. Guncar, G.; Pungercic, G.; Klemencic, I.; Turk, V.; Turk, D. Crystal structure of MHC class II-associated p41 Ii fragment bound to cathepsin L reveals the structural basis for differentiation between cathepsins L and S. *EMBO J* **1999**, *18*, 793-803, doi:10.1093/emboj/18.4.793.
3. Jurrus, E.; Engel, D.; Star, K.; Monson, K.; Brandi, J.; Felberg, L.E.; Brookes, D.H.; Wilson, L.; Chen, J.; Liles, K.; et al. Improvements to the APBS biomolecular solvation software suite. *Protein Sci* **2018**, *27*, 112-128, doi:10.1002/pro.3280.
4. Meng, E.C.; Goddard, T.D.; Pettersen, E.F.; Couch, G.S.; Pearson, Z.J.; Morris, J.H.; Ferrin, T.E. UCSF ChimeraX: Tools for structure building and analysis. *Protein Sci* **2023**, *32*, e4792, doi:10.1002/pro.4792.
5. Fojtík, L.; Fiala, J.; Pompach, P.; Chmelík, J.; Matoušek, V.; Beier, P.; Kukačka, Z.; Novák, P. Fast Fluoroalkylation of Proteins Uncovers the Structure and Dynamics of Biological Macromolecules. *J Am Chem Soc* **2021**, *143*, 20670-20679, doi:10.1021/jacs.1c07771.
6. Kukačka, Z.; Rosůlek, M.; Jelínek, J.; Slavata, L.; Kavan, D.; Novák, P. LinX: A Software Tool for Uncommon Cross-Linking Chemistry. *J Proteome Res* **2021**, *20*, 2021-2027, doi:10.1021/acs.jproteome.0c00858.
7. Martins, L.A.; Buša, M.; Chlastáková, A.; Kotál, J.; Beránková, Z.; Stergiou, N.; Jmel, M.A.; Schmitt, E.; Chmelař, J.; Mareš, M.; Kotsyfakis, M. Protease-bound structure of Ricistatin provides insights into the mechanism of action of tick salivary cystatins in the vertebrate host. *Cell Mol Life Sci* **2023**, *80*, 339, doi:10.1007/s00018-023-04993-4.
8. Götze, M.; Pettelkau, J.; Fritzsche, R.; Ihling, C.H.; Schäfer, M.; Sinz, A. Automated assignment of MS/MS cleavable cross-links in protein 3D-structure analysis. *J Am Soc Mass Spectrom* **2015**, *26*, 83-97, doi:10.1007/s13361-014-1001-1.
